# Supplementary material for: Whole-genome sequencing in 333,100 individuals reveals rare non-coding single variant and aggregate associations with height
Source: Nat Commun. 2024 Oct 3;15:8549. doi: 10.1038/s41467-024-52579-w (PMC11450065; doi:10.1038/s41467-024-52579-w)
Supplement: Supplementary file 3 — Description of Additional Supplementary Files [file 41467_2024_52579_MOESM3_ESM.pdf]

**Description of Additional Supplementary Files**

File Name: Supplementary Data 1
Description: Cohort descriptives for participants included in analyses from UK Biobank, All of Us and TOPMed

File Name: Supplementary Data 2
Description:  List of known SNPs used for adjustment in association analyses

File Name: Supplementary Data 3
Description: Novel single variants associated with height identified in discovery analysis of UK Biobank

File Name: Supplementary Data 4
Description: Genome annotations of novel single variants associated with height

File Name: Supplementary Data 5
Description: Replication results from All of Us and TOPMed of novel height-associated single variants

File Name: Supplementary Data 6
Description: Coding aggregate associations identified in UK Biobank discovery analysis of height

File Name: Supplementary Data 7
Description: Non-coding aggregate associations identified in UK Biobank discovery analysis of height

File Name: Supplementary Data 8
Description: Single variant associations (MAC>=5) contributing to the *HMGA1* upstream non-coding aggregate association

File Name: Supplementary Data 9
Description: Single variant associations (MAC>=5) contributing to the *C17orf49* downstream non-coding aggregate association

File Name: Supplementary Data 10
Description: Association statistics for non-coding aggregates in the *C17orf49* locus

File Name: Supplementary Data 11
Description: Single variant associations (MAC>=5) contributing to the *GH1* upstrean non-coding aggregate association

File Name: Supplementary Data 12
Description: Replication results from All of Us and TOPMed of the *C17orf49* and *GH1* aggregate associations

File Name: Supplementary Data 13
Description: A full list of genomic annotations tested as aggregates
